# Pilot Test on Pre-Swim Hygiene as a Factor Limiting Trihalomethane Precursors in Pool Water by Reducing Organic Matter in an Operational Facility

**DOI:** 10.3390/ijerph17207547

**Published:** 2020-10-16

**Authors:** Katarzyna Ratajczak, Aneta Pobudkowska

**Affiliations:** 1 Faculty of Environmental Engineering and Energy, Institute of Environmental Engineering and Building Installations, Poznan University of Technology, 60965 Poznań, Poland; 2 Department of Physical Chemistry, Faculty of Chemistry, Warsaw University of Technology, 00-664 Warsaw, Poland; pobudka@ch.pw.edu.pl

**Keywords:** trihalomethanes, THMs, swimming pool, pre-swim hygiene, pool water quality, disinfection byproducts

## Abstract

Pool water must be constantly disinfected. Chlorine compounds used to disinfect pools react with organic substances such as sweat, urine, and personal care products introduced into pool water by users and results in the formation of disinfection byproducts. Trihalomethanes (THM), including chloroform and dissolved organic carbon (DOC) concentrations, were quantified using a two-stage process: determining initial THM and chloroform levels; then searching for a cheap and easy-to-use method to improve water quality. The method proposed here to limit THM and DOC concentrations in water is controlled showering. At three swimming pool facilities, chloroform concentrations (13.8 ± 0.33 µg/L, 15.5 ± 0.44 µg/L, and 13.9 ± 0.06 µg/L) were below the threshold concentration of 30 μg/L. At a fourth facility, however, the chloroform concentration exceeded that threshold (40.7 ± 9.68 μg/L) when showering was not controlled. Those conditions improved after the introduction of a mandatory shower; concentrations of DOC, THMs, and chloroform all decreased. The chloroform concentration decreased to 29.4 ± 3.8 μg/L, the THM concentration was 31.3 ± 3.9 μg/L, and the DOC concentration was 6.09 ± 0.05 mg/L. Pilot tests were carried out at real facilities to determine whether the control of pre-swim hygiene was possible. The introduction of proper pre-swim hygiene limited the concentration of DOC in water and can lead to a healthier environment for everyone attending the swimming facility.

## 1. Introduction

There is a problem with water and air quality at swimming pools around the world. It is associated with the need to disinfect the pool water with chlorine compounds to protect users against viruses and bacteria as well as user-introduced organic substances. The reaction between chlorine and organic substances produces pool water disinfection by-products (DBPs). This problem was first described in 1922 by Goshorn [1] and there are more than 600 such substances [2]. Typical representatives of volatile DBPs are inorganic chloramines (mono-, di-, and tri-chloramine) and trihalomethanes (THM; chloroform, bromodichloromethane, dibromochloromethane, and bromoform) [3,4,5,6,7,8,9,10].

Research on the dynamics of volatile, gas-phase DBPs at indoor swimming facilities has identified factors affecting water and air quality in these facilities [11,12]: the concentrations of volatile compounds in pool water [13,14,15,16], the introduction of organic substances by swimmers [17,18,19,20,21], mixing of the water by swimmers [22,23] and the use of proper ventilation primarily by providing large amounts of outdoor air [14,23]. Pool water treatment also plays a factor and affects air and water quality [19]. Some research focused on changes in the water treatment system by introducing modifications such as a column with the activated carbon or with an ion-exchange deposit; this research indicated that the exchange of water led to a decrease of DBP concentrations [24,25,26,27].

Natural organic matter (NOM) of humic origin contained in clean water supplied to pools is a precursor to the formation of disinfection by-products [3,28,29]. Also, pool water contains anthropogenic pollution, including hair, saliva, urine, and personal hygiene agents that show greater reactivity towards NOM [30]. Previous studies on DBPs have also focused on their impacts on human health. Through accumulation in the cells of living organisms, they have carcinogenic, mutagenic, and teratogenic effects. These compounds can cause: asthma [24,31,32,33], upper respiratory problems [32,34,35], eye and skin irritation [6,32], cancer [36], including bladder cancer [37,38,39], and even infertility [40,41].

Pool water quality varies by country, as does the attention paid to water quality. Polish regulations have recently changed, tightening the requirements for the frequency of swimming pool water control and the scope of parameters being evaluated. They appear in the Polish Regulation of the Minister of Health of 9 November 2015 on requirements to be met by water in swimming pools [27,42]. Requirements for pool water quality in different countries have been reviewed [43]. Table 1 shows the guidelines for THM concentrations, free and combined chlorine, and pH in five European countries and the USA [44]. The maximum admissible THM concentration in many countries ranges from 20–100 μg/L.

There are approximately 700 swimming pool facilities in Poland. Those include small facilities with a sports pool basin, facilities with a sports pool and a small recreational pool, as well as large aquaparks. Pool water circuits are usually separated for each basin. A typical pool is 25 × 12.5 m with an average depth of 1.5 m.

In Poland, regulations limiting concentrations of chloroform and THM in pool water have been implemented since 2016. Despite the time that has passed since the introduction of those regulations, meeting those requirements still poses problems [45]. Maximum values, especially for chloroform, are very strict in Poland. Exceeding these limits (which are valid in Poland) occurs in many countries: U.S., France, Australia, Ireland, England, or Singapore [43] and Canada [46]. DBPs in general and chloroform in particular are global problems. Therefore, it becomes necessary to look for an easy and simple solution [46].

Since swimming pool facilities in Poland do not meet the Regulation of Ministry of Health requirements for THM concentrations, especially for chloroform, efforts were made to develop a solution for that problem. Any such solution should be simple in application, real to implement, and inexpensive to invest and operate.

### Research Goals

This study aimed to introduce showering control in selected pool facilities and to assess the impact of user behavior, including pre-swim hygiene, on the number of organic substances (precursors to THM formation) and THM formation. Such studies are rare due to the difficulty of implementing and controlling them. In running pools, it is difficult to closely control human behavior over an extended period. It was decided that the pilot studies for shower control would run for a week since coaches would be willing to control their athletes during this period. In the long run, this control would be loosened. This solution is economical in both up-front costs (soap) and long-term operation (slightly elevated use of shower water). However, if reducing concentrations of chloroform and THMs below maximum thresholds were achieved, those costs should be incurred. This study assesses pool water before the introduction of a showering control (obligation) to establish baseline levels of chloroform and THMs during standard use of the pool. Normally, swimmers do not shower before entering the pool and this observation was confirmed by lifeguards working daily at swimming pools. There is no soap in the shower area. 

Results from this research could be helpful in educational campaigns. Showering, even for short periods, might be a good solution to reduce the introduction of THM precursors to swimming pools. Limiting the precursors of THMs in water, while maintaining the concentration of free chlorine at the Polish standard range of 0.3–0.5 ppm, should allow reductions in THM concentrations. The study was divided into two stages: preliminary research-in which water status was assessed in several selected swimming pool facilities and main experiment—in selected facilities where the Regulation of the Ministry of Health requirements was not met.

## 2. Materials and Methods

### 2.1. Preliminary Research

Four swimming pool facilities in three different Polish cities and representative of typical swimming pools in Poland were selected for preliminary research. They differed in terms of usage, the number of users, and architecture, but they were chosen to represent a wide spectrum of typical swimming pools. Water treatment in each facility utilized filtration and disinfection with sodium hypochlorite in the water circuit. The pool temperature was maintained at approximately 28 °C. The amount of clean water allowed for each pool facility should result from the Regulation of the Minister of Health-30 L per person per day. The pool water exchange at each facility was conducted in different ways. Clean water was introduced after each filter backwashing process and regularly when there is a loss of water in the expansion tank or pool basin, caused by its evaporation and splashing. Most often, the recipe was implemented by periodically admitting water in the volume of its loss.

### 2.2. Characteristics of Participating Swimming Pools

#### Pool A

Pool A is a sports school facility open from 6 a.m. to 5 p.m. for school children and 5–10 p.m. for the public. The time spent in the pool was 45 min, followed by a change of pool users. Approximately 2450 people visited the pool every week. The facility contained one sports swimming pool with dimensions of 25 × 13 m. Sodium hypochlorite was used to treat the water. The water parameters during the experiment were as follows: average pool water temperature, 28.5 °C; average water temperature in the chlorine probe, 28 °C, and; average free chlorine concentration, 0.35 ppm.

#### Pool B

Pool B is a public pool open from 6 a.m. to 10 p.m. and used by various groups for any length of time. The facility has a sports pool (25 × 12 m) and a recreational pool. The water temperature was maintained at 28 °C and the free chlorine concentration range was 0.4–0.5 ppm. For this project, only the sports basin was taken into account.

#### Pool C

Pool C is a public pool open from 6 a.m. to 10 p.m. and used by various groups for any length of time. The facility has a sports pool (25 × 13 m) and a small pool with a slide. The water temperature was maintained at 28 °C, the free chlorine concentration range was 0.3–0.5 ppm. For this project, the sports basin was taken into account.

#### Pool D

Pool D is a public pool open from 6 a.m. to 10 p.m., used by various groups, and for any length of time. The facility has a sports pool (25 × 12.5 m) and a small recreational pool. The water temperature was maintained at 28 °C, the free chlorine concentration range was 0.3–0.6 ppm.

#### Sampling

Dissolved organic carbon (DOC) and THM concentrations were measured in each facility during preliminary research. In each pool, three samples for DOC and three for THMs were taken over one day. The measurements were carried out following the methodology described in Section 2.4 and Section 2.5. Water quality was assessed in terms of its temperature, combined chlorine, redox, pH, chloroform concentration, and total THMs at all four sites [47,48]. Based on those measurements, the main experiment was conducted at pools that failed to meet the Regulation of the Minister of Health requirements regarding THM concentrations. Preliminary results are described in Section 3.1. Pool A was the only pool that qualified for the main experiment.

### 2.3. Main Experiment

The main experiment took place ~two weeks after the preliminary research. Therefore, water quality testing was again carried out before the changes were introduced. Pool employees were informed about the experiment being carried out, including information on the impact of THM on the health of users and how the concentration of organic substances affects the formation of THM. Employees were informed that during the week, soap will be delivered to the pool for showers, which children and swimmers should use to wash before entering the pool or after warming up. Warming up on land before swimming is quite intense, and the swimmers work up a heavy sweat before entry. The lack of at least rinsing before entering the pool results in the introduction of a significant amount of organic substances into the pool water, i.e., THM precursors. 

In the cloakrooms and the shower area, research information was posted and a request to wash in the shower before entering the pool. In addition, coaches and trainers were asked to watch the swimmers and children so that each time before entering the water they would wash in the shower. Lifeguards working in the pool were also asked to pay attention to the need to shower. 

For this work, control of showering was defined as informing swimmers about the need to shower with soap, sending groups of children and swimmers to shower after a warm-up on land, and observation of the amount of soap used. The main experiment was carried out during the week because this was the time during which both coaches and swimmers would best adhere to the rule of washing in the shower before entering a pool. After conducting the main experiment, information on conducting the research was left, while trainers and coaches were not closely monitored on sending children and swimmers to shower. Those measurements were carried out for another week while still observing water quality parameters.

The main experiment was divided into three stages:

Stage 1: assessment of DOC and THM (including chloroform) concentrations prior to the introduction of showering control,
Stage 2: assessment of DOC and THM (including chloroform) concentrations when showering was controlled in the facility,
Stage 3: assessment of DOC and THM (including chloroform) concentrations after abandoning showering control.

### 2.4. Method for Determining THMs in Pool Water

Water samples for THM determinations were taken from the chlorine probe location. Water samples were fixed with sodium thiosulfate and stored at 4 °C until measurements were taken. Concentrations of trichloromethane (TClM), bromodichloromethane (BDClM), dibromochloromethane (DBrClM), and tribromomethane (TBrM) were determined by GC using an electron capture detector (GC-ECD) after initial concentration measurements using liquid-liquid extraction in a water/*n*-pentane system. This method was based on the Polish standard PN-EN ISO 10301: 2002 “Water quality. Determination of easily volatile halogenated hydrocarbon derivatives. Methods using gas chromatography”, with concentration ranges for each compound shown below:

- trichloromethane (chloroform): from 0.27 μg/L to 30 μg/L (300 μg/L *),
- bromodichloromethane: from 0.30 μg/L to 30 μg/L (300 μg/L *),
- dibromochloromethane: from 0.23 μg/L to 15 μg/L (150 μg/L *),
- tribromomethane: from 0.31 μg/L to 15 μg/L (150 μg/L *).

* Extending the working range results from using a 10-fold dilution of the n-pentane extract

A GC with a 6890N electron capture detector controlled by ChemStation software (Agilent Technologies, Waldbron, Germany) was used. 

### 2.5. Method of DOC Determination

Water samples for DOC determination were taken from the chlorine probe location. Water samples were stored at 4 °C until measurements were taken. For dissolved organic carbon (DOC) measurements, a TOC/TN multi NC 3100 analyzer (Endress+Hauser, Swiss) was used. Because the water sample was filtered (water passed through a 0.45 μm pore diameter filter), the TOC result corresponds to the DOC value. The determination of the carbon content was made by thermocatalytic decomposition of an 80 mL sample in the presence of an N/c catalyst at 800 °C with synthetic air as the carrier gas. 

Total carbon (TC) and total inorganic carbon (TIC) were measured. Total carbon levels reflect organic and nonionic carbon compounds as well as free carbon. TIC analysis detected inorganic carbon as carbonates and hydroxides and dissolved CO_2_. To detect the TIC, the submerged sample was metered into the TIC reactor and decomposed. CO_2_ was expelled and detected. Three TC and TIC measurements were made from one sample. Before any measurements were taken, carbon levels in ultrapure water were measured. The TOC amount was determined based on the differential from the equation: *TOC* = *TC* − *TIC*.

## 3. Results

### 3.1. Preliminary Research Results

**Figure 1:**
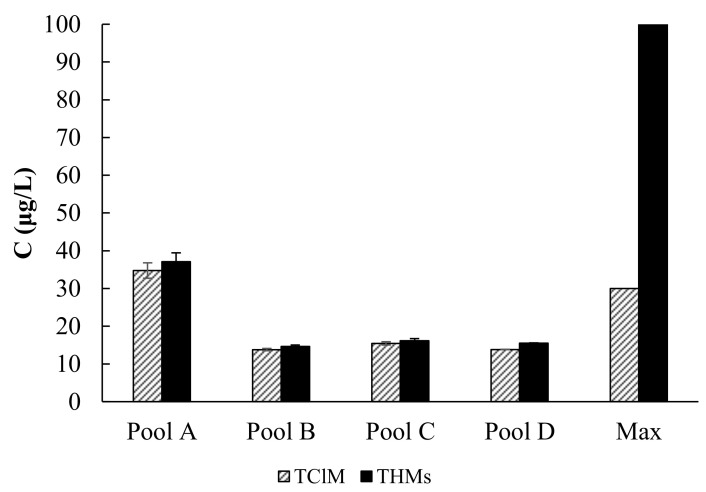
Preliminary test results—average chloroform concentrations and sum of Trihalomethanes (THMs) with standard deviation and maximum values according to the Polish Regulation of the Ministry of Health.

At each of the four pool facilities, measurements were taken over one or two days, focusing on chloroform concentrations and total THMs. Water samples were taken from the chlorine probe space to obtain values at the pool water outflow. Figure 1 shows the concentration of the sum of trihalomethanes (THMs) and chloroform (TClM) and threshold values according to the Polish ordinance of the Minister of Health in terms of total THMs (max THMs = 100 µg/L) and chloroform (max TClM = 30 µg/L). 

Results from B, C, and D, all newer than A, met Polish Regulations of the Ministry of Health requirements. The chloroform concentration, the majority component, as well as the THM sum, were well below the threshold level. Chloroform concentrations in these objects were: 13.8 ± 0.33 µg/L, 15.5 ± 0.44 µg/L, and 13.9 ± 0.06 µg/L. These values are low and relatively constant, as indicated by the standard deviation. Since pools B, C, and D met Regulation of the Ministry of Health requirements, further tests of those pools will not be taken into account. Those results indicated the pool water treatment systems were operating properly, so changes at those facilities were not necessary.

Pool A exceeded the Polish Regulation of the Ministry of Health requirements for chloroform (34.8 ± 2.03 µg/L, i.e., higher by 16%), with a maximum value of 42.5 µg/L. Pool A is 40 years old, though it has modernized the pool water treatment system. During the measurement period, two new filters worked in the water treatment system, which was installed 1.5 years earlier. The service staff indicated the pool water quality improved significantly after changing the filters though the results from this study showed that water treatment was not carried out properly, as indicated by the elevated chloroform levels. High chloroform levels have many contributing factors, but looking into options other than investing in a pool water treatment system was explored first.

### 3.2. Main Experiment Results

The elevated levels of chloroform in Pool A required additional measures to be taken to lower those levels to meet regulatory guidelines. Pool A is a school pool with a large number of children using it; group changes take place every 45 min, so it is easy to control showering in general. The coaches are school staff and were informed of the reasons for showering before entering the water. The coaches have a significant impact on the behavior of swimmers, so their participation in enforcing hygiene compliance was critical for success.

Standard pool operation in this facility requires a backwash of filters twice a week using pool water. After this, clean water is added to the pool. The pool water is changed completely once a year after the holidays. From September to June, the pool operates using the same water, replacing it after the filter backwashing process. Replacing fresh pool water or allowing more clean water [27] can solve the problem of organic substances (DOC) and trihalomethanes (THM) in water; but that is an expensive solution, so an easier and cheaper alternative was sought.

The parameters during the experiment were as follows: average pool water temperature 28.5 ± 0.4 °C, average water temperature in the chlorine probe 27.8 ± 0.2 °C, average free chlorine concentration 0.37 ± 0.07 ppm, pH 7.11 ± 0.03, redox potential 686 ± 26 mV. Detailed data are shown in Table 2.

THM and DOC concentration measurements were taken from 18 March to 4 April. Showering control occurred from March 25th to 29th. THM levels (37.4 ± 6.9 μg/L) on stage 1 were considered representative of typical THM levels and did not exceed the Polish standard for THM levels as set by Minister of Health Regulations (100 μg/L); however, the chloroform concentration, by far the largest THM component, was 35.3 ± 6.2 μg/L which exceeded regulatory levels (30 μg/L). At the same time, the amount of DOC in the water sample was 6.48 ± 0.28 mg/L. Results of measurements of THMs and DOC in conducted main experiments are shown in Table 3.

**Figure 2:**
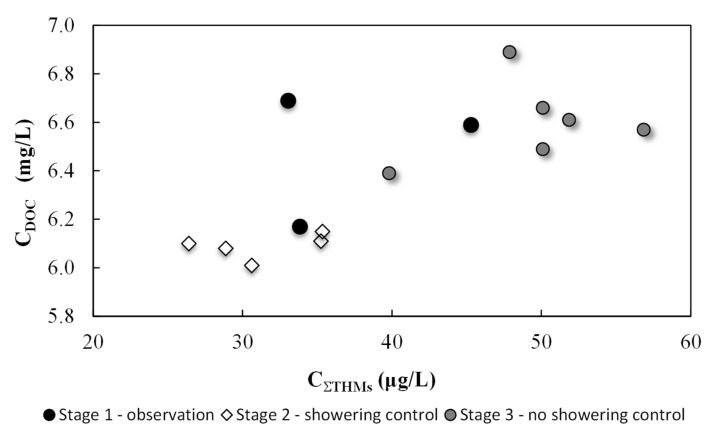
Trihalomethane (THM) concentrations in pool water as a function of organic substance concentrations (DOC) for three stages of research: observation, showering control, and with no showering control.

**Figure 3:**
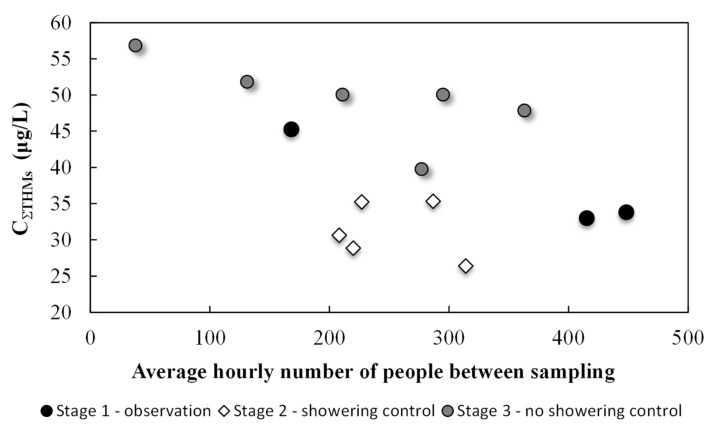
Trihalomethane (THM) concentrations as a function of pool load three stages of research: observation, showering control, and with no showering control.

**Figure 4:**
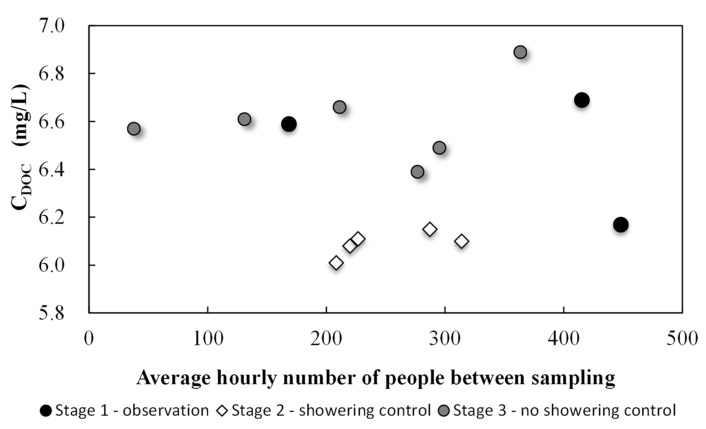
Dissolved organic carbon (DOC) concentrations depending on the pool load for three stages of research: observation, showering control, and with no showering control.

Figure 2, Figure 3 and Figure 4 show THM and DOC results divided into two parts, standard pool operations and showering control. Overall, 14 water samples were collected, including five with shower control at the pool. During the main experiment, 12 liters of soap were used during the five days with shower control. Only two liters of soap were used during the following week when showering before entering the pool was not restricted.

There was a correlation between the organic substance concentrations introduced into the water by users (DOC) and the overall THM concentration, which can be seen in Figure 4. The average THM concentration in the water during standard pool operation (Stages 1 and 3 of the experiment) was 45.2 ± 8.1 μg/L; during days with shower control that average dropped to 31.3 ± 3.9 μg/L. Showering prior to pool entry resulted in lower DOC levels. DOC values during standard pool operation (without showering control) were 6.56 ± 0.20 mg/L; with shower control, that value dropped slightly to 6.09 ± 0.05 mg/L. More stable results were found in both the THM and DOC concentrations. This indicated that increasing the awareness in pool users of the importance of thorough body washing before pool use should lead to long-term reductions in THM precursors and thus the amounts of disinfection by-products being created in the water. Though THM levels did decrease, high chloroform concentrations were still observed and exceeded threshold levels (30 μg/L). With shower control, this concentration was 29.4 ± 3.8 μg/L, and during standard pool operation, it was 42.7 ± 7.7 μg/L (exceeding the standard by 35% for standard operation). These changes saw a 27.6% decrease in THM concentrations as compared to day one levels and a 27.8% decrease in chloroform.

It should be expected that in the 3rd stage of the experiment, in which showering was not controlled, the obtained results concerning the concentration of DOC and THM should be between those obtained in the 1st and 2nd stage of the experiment. However, the THM concentrations were higher than those in stage 1. It could have been caused by the introduction of a significantly higher load of organic substances into the water than in stage 2, which could have resulted in an increased concentration of THMs. The THMs concentration is influenced, apart from the DOC concentration, by other factors, including the temperature of the water, which was slightly higher in stage 3. 

Additionally, it should be taken into account that the THM concentration is also influenced by the amount of tap water provided to the pool. What is important is the DOC concentration when showering was controlled was significantly higher than in the 2nd stage of the experiment, which contributed to the formation of THM. 

Water refilling after filter backwashing, which took place twice a week in the morning (Tuesday and Friday), resulted in lower chloroform and THM concentrations by diluting impurity concentrations. However, water refilling does not affect the concentration of organic substances (DOC). The greatest improvement was achieved in reducing DOC thanks to the introduction of controlled showering. The formation of THM is influenced by many factors, among those is the DOC concentration, hence the effects of a short-term experiment are not completely satisfactory. Nevertheless, reducing organic pollutant levels can positively affect the formation of THM. Furthermore, in the absence of shower control, a significant deterioration of water quality in the THM concentration range occurred.

## 4. Discussion

The problem of pool water disinfection by-products is universal. Different countries have established regulations regarding normal THM levels in the water. However, often these values are exceeded [43], as observed in this study. Due to this, a straightforward and economical solution to help combat increased DBP concentrations was sought. Reducing DBP levels in pool water include introducing changes in the water treatment system [19,24,26,27]. The positive impact of the total pool water exchange is also indicated, thanks to which the impurities accumulated in the water are removed [27]. Changes in water treatment technology are always difficult. It requires the use of additional water treatment sections and a significant amount of space and the funds to purchase and install. Additionally, the analyzed facilities were relatively new or had recently modernized water treatment systems; as such, additional costs were economically unjustified. Therefore, a simple and easy solution was sought, one that consisted only of providing information and soap for washing. This research showed that there are simple and cheap ways to reduce THM levels in pool water. Because the water was tested at the facility in use, THM levels differed from other results [19]; but because of this, they are also more valuable. 

Positive results were obtained because students were supervised by coaches. Encouraging pool users to take showers before entering the pool should lower DPB levels and improve the health of many pool users—not only swimmers but also lifeguards or service staff who may also suffer from diseases and health problems associated with the use of swimming pool facilities [13,35,49]. This change is not expensive [7]; however, it can be difficult to implement due to the need to change pool users’ behavior. Unfortunately, long-term monitoring of the players by the coaches (longer than a week) is not feasible, as coaches did not consider pre-swim hygiene important. In response to the necessity of proper pre-swim hygiene suggested in reports previously published [17,18,19,20,21], the tests were performed in a real, operating facility and DOC/THM reductions were monitored daily. Other groups primarily utilized laboratory measurements which, unfortunately, do not reflect real conditions. 

There is some research regarding showering and proper hygiene before pool entry—pre-swim hygiene [7,12,18,20,34,35,44]—researchers provided this recommendation but no measurements were made. Our tests examined showering habits and are described in [50]. Based on survey results [50], people take showers before entering pools to wash or to become accustomed to the water temperature. It was pointed out that pre-swim hygiene education should indicate the proper reasons for showering. However, extensive information campaigns should be carried out; this was a conclusion previously noted [51]. Posting information about rules and regulations prevailing in the pool are not observed. It is important to inform users about the impact of their behavior on their health. Based on the survey conducted by Pesonen et al. in 2013 [52], after informing users about the impact of their behavior on the processes occurring at the facility, up to 90% said they would shower before entering the pool the next time.

There are three ways to introduce DBPs into the human body: injection, dermal contact, and inhalation [36,49]. Penetration through the skin accounts for approximately 33–40% [23,33,38], while inhalation is the most common, reaching 40–67% [14,15,23,24,38,53]. Since THM concentrations in the air depend on the concentrations in the water, limiting levels of those compounds in water should be examined [11,20,54,55]. For these reasons, permissible THM levels in pool water are strictly controlled.

Pilot tests were carried out at several operating facilities and its importance stems from the fact it showed that it is possible to apply conclusions obtained in the laboratory. This type of research is not often conducted on-site due to its uncertain nature. Controlling the behavior of people is difficult; therefore, there is a relative dearth of research data that certainly requires additional study, especially since these tests showed significant potential for improving swimming pool water quality.

Showering control and proper pre-swim hygiene reduced DOC levels in water (without a large monetary investment), which lead to a THM decrease. Due to their volatility, this procedure will also improve pool air quality. Thanks to the obligatory shower of users, THM levels were reduced by 27% and significantly improved pool water quality. These results came close to the maximum chloroform threshold level as set by legal regulations (30 μg/L).

These experiments should be repeated and transferred to other pool facilities; though preliminary results are promising and suggested an economical way to reduce DBP levels. However, it is difficult to find a pool where regular groups of users participate in the activities and changes occur at specific intervals. Only then is it possible to control showering. When people change continuously rather than in groups, it is more difficult to encourage them to shower. 

## 5. Conclusions

The pilot studies were aimed at assessing whether it is possible to carry out tests on the influence of pre-swim hygiene on the quality of swimming pool water in a normal operating facility. Pre-swim hygiene is important but difficult to control. It is necessary to make people aware that their behavior is a key component affecting their indoor environment.

This study confirmed that showering before entering the pool improved water quality in a simple and economic way. Controlling user behavior by introducing a shower requirement reduced THM levels in pool water by 27%.

The obtained results could be used in educational campaigns, in which people should be educated about the problem of water quality in swimming pools by indicating that an application of the basic principles such as pre-swim hygiene will keep the swimming pool environment much healthier.

## Figures and Tables

**Table 1:** Pool water quality requirements in five European countries and the United States.

Country	Maximum Concentration		Free Chlorine	Combined Chlorine	pH
	(μg/L)		(mg/L)	(mg/L)	(−)
Germany	20	chloroform	0.3–0.6	0.2	6.5–7.6
Switzerland	30	THMs	<3.0	0.2	7.2–7.8
Belgium	100	chloroform	0.3–1.4	0.6	6.9–7.7
United Kingdom	100	THMs	1.0–2.0	0.2	−
Poland	100	THMs	0.3–0.6	0.3	6.5–7.6
	30	chloroform			
United States	80 (drinking water)	THMs	1–5 (2–4)		7.4–7.6

**Table 2:** Water and air parameters and the number of pool users between the collected samples in Pool A during the main experiment.

Experiment Stage	Date of Water Sample Collection	At the Sampling Point				Air Temperature	Average Hourly Number of People between Sampling
		Water Temperature	Free Chlorine **	Redox	pH		
		(°C)	(ppm)	(mV)	(-)	(°C)	
Stage 1 observation	Mar 18 8:15 a.m.	27.7	0.40	682	7.10	25.0	168
	Mar 19 3:00 p.m.	27.7	0.25	645	7.09	25.5	448
	Mar 21 5:30 p.m.	27.8	0.44	678	7.10	25.0	415
Stage 2 showering control	Mar 25 3:00 p.m.*	27.5	0.29	699	7.10	25.1	221
	Mar 26 3:00 p.m.*	27.7	0.33	677	7.13	24.9	315
	Mar 27 2:50 p.m.*	27.8	0.30	730	7.12	25.4	227
	Mar 28 2:30 p.m.*	27.8	0.30	709	7.12	25.1	287
	Mar 29 10:00 a.m.*	27.7	0.38	714	7.07	24.9	208
Stage 3 no showering control	Mar 30 4:00 p.m.	28.2	0.39	692	7.10	25.6	211
	Mar 31 4:00 p.m.	28.0	0.40	674	7.07	25.1	38
	Apr 01 3:00 p.m.	27.7	0.50	686	7.02	26.1	363
	Apr 02 1:30 p.m.	28.0	0.45	690	7.10	26.1	277
	Apr 03 3:00 p.m.	27.8	0.33	640	7.11	26.1	296
	Apr 04 11:00 a.m.	28.2	0.40	650	7.10	26.5	132
	Average	27.8 ± 0.2	0.37 ± 0.07	683.3 ± 26	7.1 ± 0.03	25.5 ± 0.53	350 people/day

* control of showering; ** max free chlorine 0.5 ppm.

**Table 3:** Pool A DOC and THM concentrations in the main experiment.

Experiment Stages	Date of Water Sample Collection	DOC	TClM	BrDClM	DBrClM	TBrM	∑THM
		(mg/L)	(μg/L)	(μg/L)	(μg/L)	(μg/L)	(μg/L)
Stage 1 observation	Mar 18 8:15 a.m.	6.59	42.5	2.79	<0.31	<0.30	45.3
	Mar 19 3:00 p.m.	6.17	32.3	1.53	<0.31	<0.30	33.8
	Mar 21 5:30 p.m.	6.69	31.2	1.83	<0.31	<0.30	33.0
Stage 2 showering control	Mar 25 3:00 p.m.*	6.10	26.6	2.27	<0.31	<0.30	28.9
	Mar 26 3:00 p.m.*	6.10	24.9	1.50	<0.31	<0.30	26.4
	Mar 27 2:50 p.m.*	6.11	33.2	2.04	<0.31	<0.30	35.2
	Mar 28 2:30 p.m.*	6.15	33.3	2.04	<0.31	<0.30	35.3
	Mar 29 10:00 a.m.*	6.01	28.8	1.83	<0.31	<0.30	30.6
Stage 3 no showering control	Mar 30 4:00 p.m.	6.66	47.4	2.67	<0.31	<0.30	50.1
	Mar 31 4:00 p.m.	6.57	53.9	2.95	<0.31	<0.30	56.9
	Apr 01 3:00 p.m.	6.89	45.1	2.78	<0.31	<0.30	47.9
	Apr 02 1:30 p.m.	6.39	37.2	2.59	<0.31	<0.30	39.8
	Apr 03 3:00 p.m.	6.49	46.5	3.17	0.39	<0.30	49.7
	Apr 04 11:00 a.m.	6.61	48.2	3.23	0.4	<0.30	50.5

*showering control; DOC—dissolved organic carbon; TClM—trichloromethane (chloroform); BrDClM—bromodichloromethane; DBrClM—dibromochloromethane; TBrM—tribromomethane; ∑THM—sum of trihalomethane.
